# A dual-functional Embolization-Visualization System for Fluorescence image-guided Tumor Resection

**DOI:** 10.7150/thno.39700

**Published:** 2020-03-15

**Authors:** M. Martin Jensen, Zachary B. Barber, Nitish Khurana, Kyle J. Isaacson, Douglas Steinhauff, Bryant Green, Joseph Cappello, Abigail Pulsipher, Hamidreza Ghandehari, Jeremiah A. Alt

**Affiliations:** 1 Department of Bioengineering, University of Utah, Salt Lake City, UT, 84112 USA.; 2 Utah Center for Nanomedicine, Nano Institute of Utah, University of Utah, Salt Lake City, UT, 84112 USA.; 3 Department of Pharmaceutics and Pharmaceutical Chemistry, University of Utah, Salt Lake City, UT, 84112 USA.; 4 Department of Surgery, Division of Otolaryngology-Head and Neck Surgery, University of Utah School of Medicine, Salt Lake City, UT 84113.

**Keywords:** Embolic, Image-Guided Surgery, Indocyanine Green, Silk-Elastinlike Protein Polymer

## Abstract

**Rationale**: Intraoperative bleeding impairs physicians' ability to visualize the surgical field, leading to increased risk of surgical complications and reduced outcomes. Bleeding is particularly challenging during endoscopic-assisted surgical resection of hypervascular tumors in the head and neck. A tool that controls bleeding while marking tumor margins has the potential to improve gross tumor resection, reduce surgical morbidity, decrease blood loss, shorten procedure time, prevent damage to surrounding tissues, and limit postoperative pain. Herein, we develop and characterize a new system that combines pre-surgical embolization with improved visualization for endoscopic fluorescence image-guided tumor resection.

**Methods**: Silk-elastinlike protein (SELP) polymers were employed as liquid embolic vehicles for delivery of a clinically used near-infrared dye, indocyanine green (ICG). The biophysical properties of SELP, including gelation kinetics, modulus of elasticity, and viscosity, in response to ICG incorporation using rheology, were characterized. ICG release from embolic SELP was modeled in tissue phantoms and via fluorescence imaging. The embolic capability of the SELP-ICG system was then tested in a microfluidic model of tumor vasculature. Lastly, the cytotoxicity of the SELP-ICG system in L-929 fibroblasts and human umbilical vein endothelial cells (HUVEC) was assessed.

**Results**: ICG incorporation into SELP accelerated gelation and increased its modulus of elasticity. The SELP embolic system released 83 ± 8% of the total ICG within 24 hours, matching clinical practice for pre-surgical embolization procedures. Adding ICG to SELP did not reduce injectability, but did improve the gelation kinetics. After simulated embolization, ICG released from SELP in tissue phantoms diffused a sufficient distance to deliver dye throughout a tumor. ICG-loaded SELP was injectable through a clinical 2.3 Fr microcatheter and demonstrated deep penetration into 50-µm microfluidic-simulated blood vessels with durable occlusion. Incorporation of ICG into SELP improved biocompatibility with HUVECs, but had no effect on L-929 cell viability.

**Principle Conclusions**: We report the development and characterization of a new, dual-functional embolization-visualization system for improving fluorescence-imaged endoscopic surgical resection of hypervascular tumors.

## Introduction

Distinguishing tumor margins from normal tissue in surgery is challenging, as surgeons heavily rely on haptic feedback during surgical dissection. The loss of the sense of touch and proprioception is especially problematic during endoscopic surgery in the sinonasal cavity, as tumor margins can only be assessed via visualization through an endoscope and digital manipulation with rigid instruments. Further, maintaining good visualization of the surgical field and being able to identify normal tissue versus the tumor is paramount for safe and successful oncological surgery. Endoscopic surgical resection of hypervascular tumors can be challenging due to 1) reduced visibility from bleeding and 2) loss of haptic feedback due to the inability to palpate tissue, leading to difficulties in identifying normal tissue from tumor tissue, increasing the risk of surgical complications and suboptimal gross tumor resection. These challenges are compounded during the resection of hypervascular tumors in the sinonasal cavity, where significant intraoperative bleeding can rapidly obscure the visual field due to the limited space and ability to access the tumor in this unique surgical corridor. Juvenile nasopharyngeal angiofibroma (JNA) are particularly difficult to remove, due to their location in the sphenopalatine foramen and proximity to critical structures such as the trigeminal nerve, internal and external carotid, optic nerve, orbit, and the brain 1. As surgery is currently the most common and effective form of treatment for JNAs, clear and defined margins are critical for achieving optimal outcomes. The development of new methods that can improve intraoperative visualization by reducing bleeding while enhancing demarcation of tumor margins could greatly improve safety and oncologic outcomes in these complex cases.

Fluorescence-based image-guided surgery has shown great potential to intraoperatively detect malignant tissue in endoscopic and robotic surgeries and distinguish tumor margins 2-4. Near infrared (NIR) imaging with fluorescent NIR contrast agents utilize wavelengths in the range of 700-900 nm. NIR imaging minimizes background autofluorescence and allows for the greatest transmission of light within tissues. However, rapid dilution after administration and short circulation time result in low accumulation of dyes within the desired tissues. Embolization provides a unique opportunity to overcome both of these short-comings, by locally delivering a higher concentration of a fluorescent dye, thereby reducing its clearance from the tumor by occluding blood flow.

Pre-surgical embolization is currently practiced for a variety of tumors in the head and neck in order to reduce intraoperative bleeding during surgical resection 5. Reduced intraoperative bleeding can help decrease operative time, improve visualization of the surgical field, decrease risk of surgical complications in adjacent tissues, and decrease the risk of tumor recurrence 5,6. Current embolic materials are ill-suited for fluorescent marking due to poor tumor penetration and incompatibility with clinically approved dyes 7,8. Particle-based clinical embolics (*i.e.*, microspheres and gelatin foams) can efficiently block tumor blood supply, but these materials fail to deeply penetrate the tumor vasculature 7-10. Current clinically available drug eluting microspheres use electrostatic interactions to load and mediate the release of loaded therapeutic materials. These drug eluting beads are currently used only for the delivery of positively charged drugs such as doxorubicin and irinotecan 7. Liquid embolic agents, such as acrylic glues (Truefil™), can only penetrate blood vessels to a depth of 0.5 mm and only spontaneously solidify when polar or charged materials are added. New embolic delivery vehicles are needed to expand the physician's armamentarium and allow for the delivery of hydrophilic, anionic, and biological therapies to tumors. An ideal embolic agent for pre-surgical embolization should be capable of 1) delivering a marker to tumors, 2) deeply penetrating into and occlude vasculature, and 3) release the majority of its payload in accordance with surgical procedural timing.

Indocyanine green (ICG) has shown promise in head and neck surgical procedures for a variety of cancers 11. ICG binds avidly to albumin and other globular proteins that naturally extravasate into tissue at the capillary level 12. ICG accumulates preferentially in tumor tissue due to poor lymphatic recycling of albumin and other blood proteins compared with healthy tissues. This difference in clearance can create a well-defined boundary that corresponds to tumor margins 12,13. Rapid dilution after intravenous administration and rapid clearance by the liver, half-life of only 3-5 minutes, limit ICG's ability to accumulate in tumors and successfully demarcate tumor margins. Only approximately 0.05% of an ICG dose typically remains within the tumor by the time of surgery 14. This challenge could be overcome by locally delivering ICG and restricting blood flow within a tumor.

Silk-elastinlike protein (SELP)-based embolics have the potential to locally deliver ICG while achieving effective embolization. SELPs are genetically engineered protein-based polymers that combine the temperature-responsive solubility of elastin and the physical strength of silk 15-17. The ability to control SELPs at a molecular level allows the precise tailoring of protein structure to function in a predictable and exquisitely tunable fashion. SELPs dissolved in saline are highly biocompatible and have mechanical properties for use as an *in situ* gelling embolic 7,18,19. SELPs represent an innovative solution to overcome the shortcomings of current clinical tools for embolizing hypervascular tumors. These embolics can deeply penetrate the tumor before rapidly transitioning to form a solid gel, use a biocompatible aqueous solution, and can carry up to 50 mg/mL of loaded compounds 20. In this manuscript, we report the development of a dual-function SELP-based embolization-visualization system that has the potential to reduce intraoperative bleeding, while simultaneously delivering ICG to fluorescently demarcate tumor margins. We characterized the biophysical properties of SELP in response to ICG incorporation and penetration efficiency in phantom agar tissues. The dual-functionality of the SELP-ICG system was then evaluated in a microfluidic model of tumor vasculature. To assess the biocompatibility of this new system, we tested the viability of model mammalian cell lines in response to SELP-ICG incubation.

## Materials and Methods

### Materials

**Figure 1:**
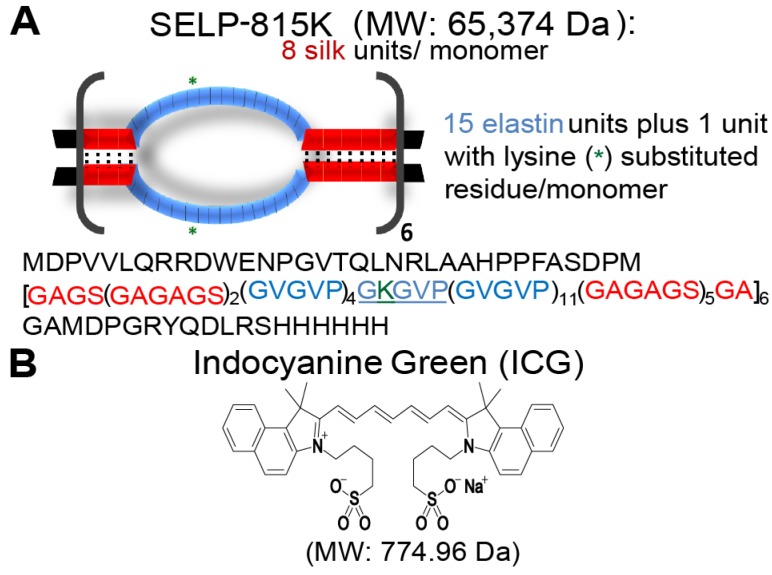
** Structures of indocyanine green and SELP 815K. A)** Illustration of silk-elastinlike protein polymer (SELP) 815K structure. The single letter amino acid code for the protein polymer is listed below in the graphic. MW: Molecular Weight.** B)** Chemical Structure of indocyanine green (ICG).

SELP 815K was expressed in *Escherichia coli* and purified, characterized, and shear-processed as previously described (Figure 1A) 18,20,21. ICG sodium salt (Figure 1B) was obtained from Sigma Aldrich (St. Louis, MO). Dulbecco's Phosphate Buffered Saline (PBS), Express Enzyme with no phenol red, trypan blue, and Fetal Bovine Serum (FBS) were obtained from ThermoFisher Scientific (Waltham, MA). Triton X, sodium azide, Endothelial Cell Growth Medium (ECGM), and bovine serum albumin (BSA) were obtained from Sigma Aldrich (St. Louis, MO). FD&C red dyes 40 and 3 were obtained in a premixed solution (McCormick, Hunt Valley, MD) to serve as visual indicators. L-929, murine fibroblasts and human umbilical vein endothelial cells (HUVECs) were obtained from the American Type Culture Collection (ATCC) (Manassas, VA).

### Effect of ICG on SELP hydrogels

To evaluate the effect of ICG on the swelling behavior of SELP hydrogels, frozen SELP 815K 12% (wt/wt) was thawed, mixed with 0, 0.1, 1.0, 5.0, and 10.0 mg/mL ICG, and then incubated for 12 hours at 37°C in tuberculin syringes. These concentrations spanned the range above and below concentrations used clinically for ICG injections. The end of the syringe was removed, and the SELP-ICG mixture was cut into 20 ± 1 µL cylindrical samples (~3.5 mm in diameter, ~2 mm in height) and weighed. These were placed into 1.0 mL of PBS and incubated for 2 weeks at 37°C. Samples of SELP with and without 0.5 mg/mL ICG were flash frozen in liquid nitrogen and lyophilized at -50°C and <0.06 mbar for 4 days on Labconco lyophilizer (Kansas City, MO). Free ICG and ICG incorporation into the gels were quantified using absorbance detection at 780 nm with a SpectraMax M2 Spectrophotometer (Molecular Devices, Sunnyvale, CA). The swelling ratios and soluble fractions were calculated as previously described 22. Scanning electron microscopy (SEM) was performed on a FEI Quanta 600F (ThermoFisher Scientific, Waltham, MA) as previously described to evaluate the possible effects of ICG incorporation on SELP microstructure morphology 23. Pore size was calculated using ImageJ software version 1.52a (National Institute of Health, Bethesda, MD) by thresholding SEM images to mark pore structures and then using the built in particle analyses function.

### ICG Release from SELP

A release study was conducted to determine the effect of ICG concentration on its release profiles from the hydrogels. SELP-ICG was loaded with concentrations of dye appropriate for imaging after local delivery (0.005, 0.05, and 0.5 mg/mL) by directly mixing the powdered dye into the polymer solution. Beginning with 20 μL of SELP 815K 12% (wt/wt) (n=5), a concentration that has previously demonstrated embolic potential, was injected into a 1 mL vial through a 30g needle with a chilled Hamilton syringe 18. SELP was then incubated at 37°C for 12 hrs. To begin the release study, 1 mL of prewarmed (37°C) PBS supplemented with 50 mg/mL of bovine serum albumin (BSA) was added to the samples. As a control for ICG stability, 1 mg/mL of ICG dissolved in release media was used and treated identically to samples. Prior to injection, the release media was prewarmed to 37°C. At designated time points (0, 0.12, 0.25, 0.5, 1, 3, 6, 12, 24, 36, and 48 hrs), 100 μL of media was removed and replaced from each vial, added to a 96-well plate, and assayed at 780 nm on a SpectraMax M2 spectrophotometer. To analyze the release profile of ICG from the SELP hydrogels, the data were fit to the Korsmeyer-Peppas model (see Equation 1) 24.


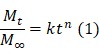


### Viscoelastic properties of SELP Embolic loaded with ICG

The following embolic properties are achieved with SELP: an initial injectable viscosity, a rapid transition to an occlusive gel after injection within the target vasculature, and the ability to achieve a modulus capable of resisting intraarterial pressures. To quantify these features, the viscoelastic properties were evaluated using rheology as previously described 18,23. Samples were analyzed using a temperature ramp from 18 to 37°C (5.8°C/min) and a 20-mm, 4° cone geometry on a TA AR550-Stress Controlled Rheometer (New Castle, DE). This was followed by a 3-hr oscillatory time sweep at 37°C, 0.1% strain, and an angular frequency of 6.283 rad/s. Gelation and the potential for phase separation were evaluated using a tilt test. SELP 815K 12 wt% (400 µl) with 0.5 mg/mL of ICG was cooled in a glass chromatography vial (ThermoFisher Scientific, Waltham, MA), on ice. The vials were then hermetically sealed and placed into a 37°C water bath in an upright position for 1, 2, 3, 5, 10, 15, 30 and 60 min. At each time point, each vial was briefly removed and photographed after being tilted at 90°. The images were globally white-balanced and cropped to remove excess background.

### ICG release and diffusion behavior in tissue phantoms

Tissue-mimicking agar phantoms were used to measure ICG release from SELP and its diffusion behavior after simulating endovascular embolization. The agar phantoms were generated by dissolving 35 g/L of BD Bacto agar in deionized water prior to being autoclaved 25. The solution was then cooled to <50°C, to which 200 mg/L of sodium azide and 35 g/L of BSA were added to respectively prevent bacterial growth and add a structural protein component. The phantom molds were cast in Cellstar® 6-well cell culture plates (Greiner, Austria) with segments of polyethylene (0.7-mm outer diameter) (Cole-Parmer, Vernon Hills, IL) that were threaded through pre-punched holes into each well. This process created a small void that ran through the center of each phantom to mimic the size of a blood vessel running through tissue that could be selectively embolized using clinical microcatheters. Each well was filled with 15 mL agar and allowed to gel at room temperature for 24 hrs. ICG was imaged within the phantoms using a 5 second exposure time and 780 nm excitation and an 831 nm emission with the Spectrum In Vivo Imaging System (IVIS) (Caliper Life Sciences, Massachusetts, USA). ICG (0.5 mg/mL) release from SELP and diffusion behavior was tested in triplicate for each phantom type. A non-SELP control, 0.5 mg/mL ICG in 50 mg/mL BSA in PBS, was tested to evaluate if SELP impaired the partitioning of ICG into the simulated tissue in each phantom type. ICG diffusion behavior was quantified by measuring its diffusional distances from the void over 48 hrs. A MATLAB script (The Mathworks, Inc., Natick, MA) was used to calculate the mean intensity of the fluorescent signal at varying distances from the center of the gel in images acquired on the IVIS. The radius at which the signal reached 10% of the maximum fluorescence intensity for each gel was designated as the visual front of diffusing dye.

### Correlation of ICG imaging between IVIS and a clinical endoscopic system

A Karl Storz 4mm × 18cm ICG endoscope (Karl Storz, Tuttlingen, Germany) attached with a Power LED light source with fiber optic ICG cable and Karl Storz Image 1s Video System with ICG High Def Camera Head (Karl Storz, Tuttlingen, Germany) was used for imaging the ICG at different concentrations. ICG solution was prepared by mixing 25 mg of ICG in 1 mL of water, resulting in a 25 mg/mL solution. The solution was diluted by half in subsequent wells of a 96-well plate and the last well was filled with only water (negative control). The endoscope's imaging head was held 4 mm above the surface of the 96-well plate to acquire images. Fluorescent light was captured by the camera and shown as blue. The intensity of the fluorescent-light was quantified using the Zen Lite Blue software version 2.6 (Zeiss, Oberkochen, Germany). A fixed area of equal size for each well was selected and the fluorescence was quantified by taking arithmetic mean intensity of blue contribution for that particular rein pixels. IVIS was used to image ICG at different concentrations. Similar solutions of ICG were prepared and loaded in a 96-well plate. The same 96-well plate imaged with the Karl Storz endoscope was set on the sample stage. Living Image® Software (PerkinElmer, Massachusetts, USA) was used to take images of the 96-well plate. All the settings for acquiring the image were done in the software (Exposure time - 1.50 seconds; Field of view - 12.5 cm; F/stop (aperture) - 2; Pixel Binning - medium). An overlay image combination of photographic and fluorescent image was recorded. The region of interest (ROI) tool was used to perform quantification of the surface intensities. Equal size ROIs were drawn on each well of the plate and the radiant efficiency was recorded by the software for each defined ROI. Signal from the negative control well was subtracted to correct for background signal. The various radiant efficiencies were compared to different concentrations of ICG and results were plotted. Linear regression statistical analysis was also performed using GraphPad Prism 5 (GraphPad Software, San Diego, CA) to find correlations between the endoscopic fluorescent imaging and IVIS radiant efficiencies of ICG.

### Embolization of microfluidic models of tumor vasculature

Highly selective embolization requires the ability to pass through clinical microcatheters with small diameters and then selectively occlude target vasculature. To simulate tumor vasculature, a microfluidic device was designed based upon the Murray-Hess Law as previously described 18. Devices representing tortuous conduits and of branching networks of tumor vasculature were constructed using Sylgard 184 silicone elastomer (Dow Corning, Midland, MI). Silicone was prepared per the manufacturer's instructions, de-aerated, poured over the mold, and cured at 153°C. After curing, the mold was removed, and the silicone was plasma-bonded to a glass microscope slide using an Enercon Dyne-A-Mite Air Plasma Surface Treater (Enercon, Menomonee Falls, WI). During testing, three microfluidic devices were connected in parallel to a central syringe pump. A 20-mL syringe was filled with PBS with red food color (McCormick & Company, Inc., Baltimore, MD) for visualization. Saline was pumped through the devices at a flow rate of 0.63 mL/s to achieve a pressure of 40 mmHg, modeling rates and pressures found within blood vessels of similar cross-sectional area 26. SELP with 0.5 mg/mL ICG was injected into the microfluidic tumor model devices using 2.3-Fr, 110-cm microcatheter (Merit, South Jordan, UT) submerged in a 37°C water bath to simulate clinical procedures. The second and third devices, representing collateral vascular beds that feed nonmalignant tissue around the tumor, were connected in parallel to the test chip in order to evaluate the potential off target embolization due to retrograde flow and provide an alternate path of flow after the occlusion of the tumor vasculature. After each test, the embolized device was replaced and the other chips investigated for evidence of occlusion using IVIS as described in the diffusion study. If no occlusion was observed the collateral chips were reused. This experiment was replicated 3 times.

### Cytotoxicity of SELP ICG

L-929 and HUVEC were selected for use in assessing the cytotoxicity of the SELP-ICG embolic based upon their utility with respect to regulatory testing and relevance to the intended application of the device. L-929 cells are commonly used for FDA testing of contact cytotoxicity of medical devices, such as embolics. HUVEC represents a human cell line, another common cell that is also frequently used for evaluating cytotoxicity. Additionally, HUVEC represents vascular endothelial cells that embolic SELP will be in close contact with during *in vivo* administration. L-929 fibroblasts were grown with DMEM: F12 (1:1) media, supplemented with 10% FBS, and HUVECs were grown in ECGM. The cells were grown in T-75 flasks at 37°C with 5% CO_2_ and passaged at 80-95% confluency. Cells were suspended using TrypLE™ Express Enzyme with no phenol red according to the manufactures protocol. The viability of cells was assessed using 0.4% trypan blue stain using a Countess Automated Cell Counter (ThermoFisher Scientific, Waltham, MA). L-929 cells were seeded into new T75 flasks with 3x10^5^ to 6x10^5^ viable cells. HUVECs were seeded into new T-75 Flasks with 1x10^5^ to 7x10^5^ cells for each passage. Only cell cultures with greater than 90% viability, typically >95%, were used in assays. Cells were seeded into 96-well plates for testing before their 6^th^ passage. SELP 815K 12% (wt/wt) with 0.5 mg/mL ICG and PBS with ICG 0.5 mg/mL were used as test samples and serially diluted to generate standard concentration curves. Viability was measured after 24 hours using a Cell Counting Kit (CCK)-8 assay kit (Dojindo, Kumamoto, Japan). Higher concentrations of gel were observed to form solid gels or particles during incubation under culture conditions. No treatment and 1% Triton-X were used as positive and negative controls, respectively. LD_50_ values were determined by fitting the data to a Hill plot with a variable slope using GraphPad Prism 5.0.

### Statistics

The data were collected and processed using Excel (Microsoft, Redmond, WA), and statistical and regression analyses were performed using GraphPad Prism 5.0. Outliers were identified using a Grubb's Test and excluded from cytotoxicity testing. The data analyzed in this study were assumed to be parametric in nature. Paired sets of data were analyzed using the Student's T-test for paired sets of data and one-way analysis of variance (ANOVA) with a post-hoc Bonferroni multiple comparison test to compare data sets with 3 or more groups. Least squares regression was used to assess linear correlations in the data. A p-value of less than 0.05 was used as the threshold for statistical significance.

## Results

### Effect of ICG on SELP hydrogels

**Figure 2:**
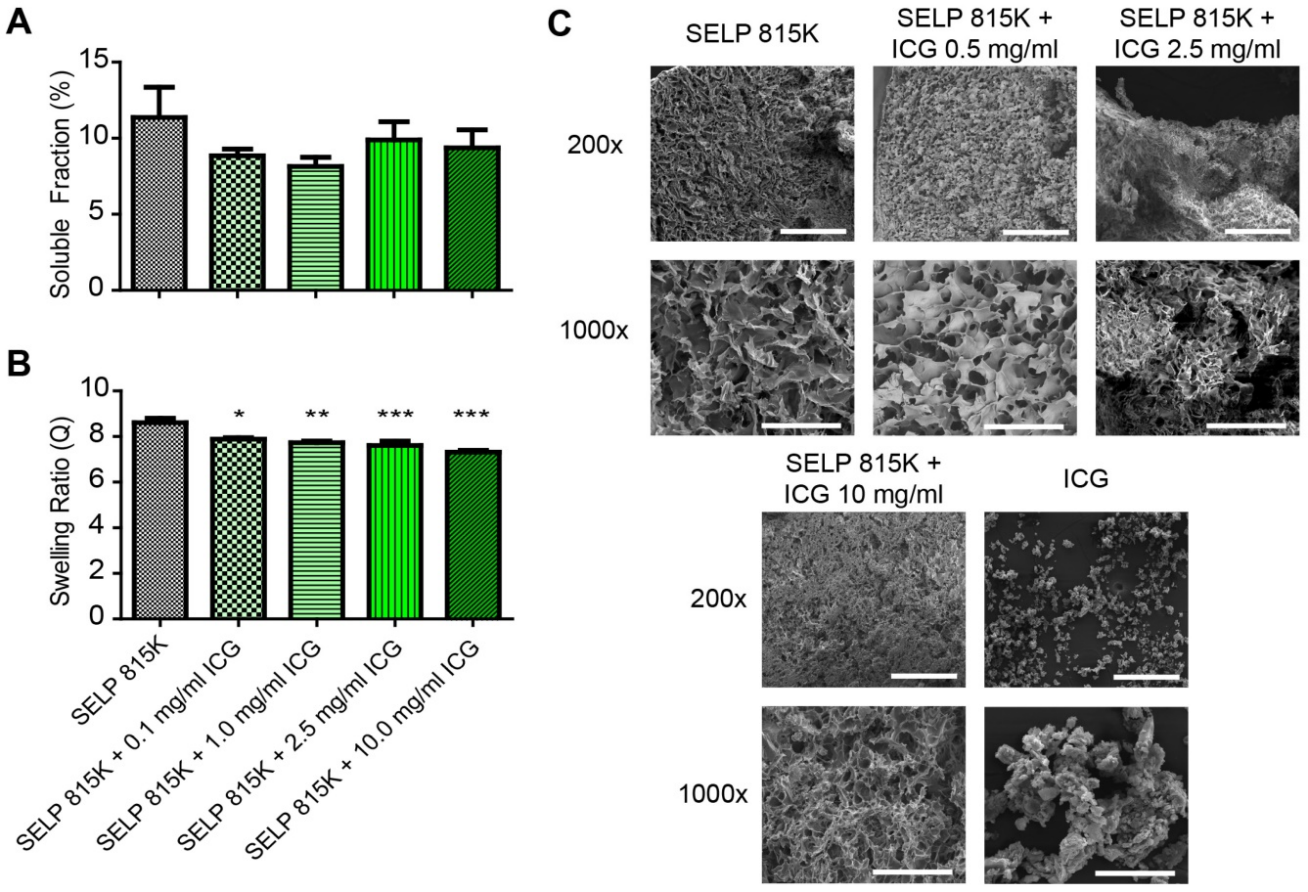
** Effect of ICG on SELP hydrogel properties.** The **A)** soluble fractions and **B)** swelling ratios of SELP 815K hydrogels loaded with ICG. The data represent the mean ± st. dev. of n=6 samples. **C)** SEM images demonstrating lyophilized SELP microstructures with varying ICG concentrations. The scale bars represent 200 µm and 50 µm for the 200x and 1000x, respectively. *:p<0.05, **:p<0.01, ***:p<0.001.

ICG increases SELP 815K polymer interactions, resulting in the formation of a denser hydrogel matrix. The soluble fraction of SELP is decreased by the addition of ICG (Figure 2A). The swelling ratio of the SELP hydrogels significantly decreases with increasing concentrations of ICG. At 0.1 mg/mL ICG, the swelling ratio is decreased 8.2% and at 10 mg/mL ICG, the swelling ratio decreased by 15.2% (Figure 1B). While statistically significant, the relatively modest decrease in swelling ratio should not impact the gel's ability to occlude blood flow. Both of these trends indicate increased polymer-polymer interaction due to the presence of ICG. SEM imaging revealed visible changes in SELP microstructure after ICG incorporation (Figure 2C). The matrix became appreciably denser with smaller voids and thicker partitions. SELP matrices with 0, 0.5, 2.5, and 10 mg/mL of ICG had pores with average areas of 21 ± 38, 15 ± 23, 4 ± 24, and 25 ± 75 µm^2^, respectively. The pores in all of the matrices were irregular and existed at multiple size scales resulting in degree of variation in measured pore sizes (Figure 1C). Taken together, the addition of ICG to SELP tends to increase polymer interactions, leading to the formation of denser hydrogel matrices, which can alter viscosity and release kinetics.

### ICG release from SELP

**Figure 3:**
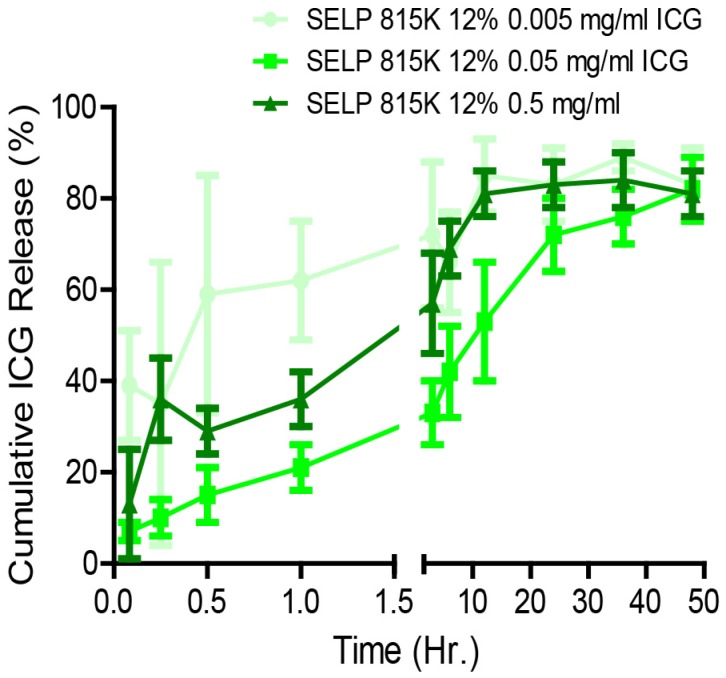
** Effect of concentration on ICG release from SELP hydrogels.** The data represent mean ± st. dev. of n=6 samples.

Figure 2C demonstrates that ICG incorporation altered the microstructure of SELP in a concentration-dependent fashion. To investigate if these structural changes had an effect on ICG release, we evaluated the release kinetics by varying ICG-SELP compositions. ICG concentrations were selected to maximize the potential fluorescent signal in the context of tumor vasculature embolization and self-quenching behavior of ICG. The materials were injected through a 30g needle and formed solid cohesive droplets at the bottom of the vials that did not phase separate. These features are necessary for the material to be able to be injected endovascularly, maintaining a high enough concentration to gel, and occlude the whole vascular lumen to prevent blood flow after administration. Burst release was greatest for 0.005 mg/mL group, which released 39 ± 12% of the ICG payload within 5 minutes of injection (Figure 3). However, the relative burst release for higher concentrations of ICG was significantly reduced. The burst release was only 7 ± 2% for the 0.05 mg/mL ICG group. The release profiles for 0.5, 0.05, and 0.005 mg/mL of ICG were consistent with first-order release kinetics and had n values ranging from 0.151 ± 0.029 to 0.417 ± 0.011 (mean ± standard error) in the Korsmeyer-Peppas model, indicating that quasi-Fickian diffusion was mediating the release of ICG (see Figure S1). The 0.5, 0.05 and 0.005 mg/mL ICG gels released 84 ± 6.0%, 72 ± 8.0%, and 83 ± 8.0%, respectively, of their payloads within 24 hrs, which is functional for pre-surgical embolization procedures as they are performed the day prior to tumor resection. The cumulative mass of ICG (mg of ICG) released by each gel at 24 hr. was significantly different (p<0.001). Based upon these results and considering the anticipated volume of distribution of the dye during intravascular release within a tumor, SELP loaded with 0.5 mg/mL of ICG was selected for testing as an embolic material.

### Viscoelastic properties of SELP embolic loaded with ICG

**Figure 4:**
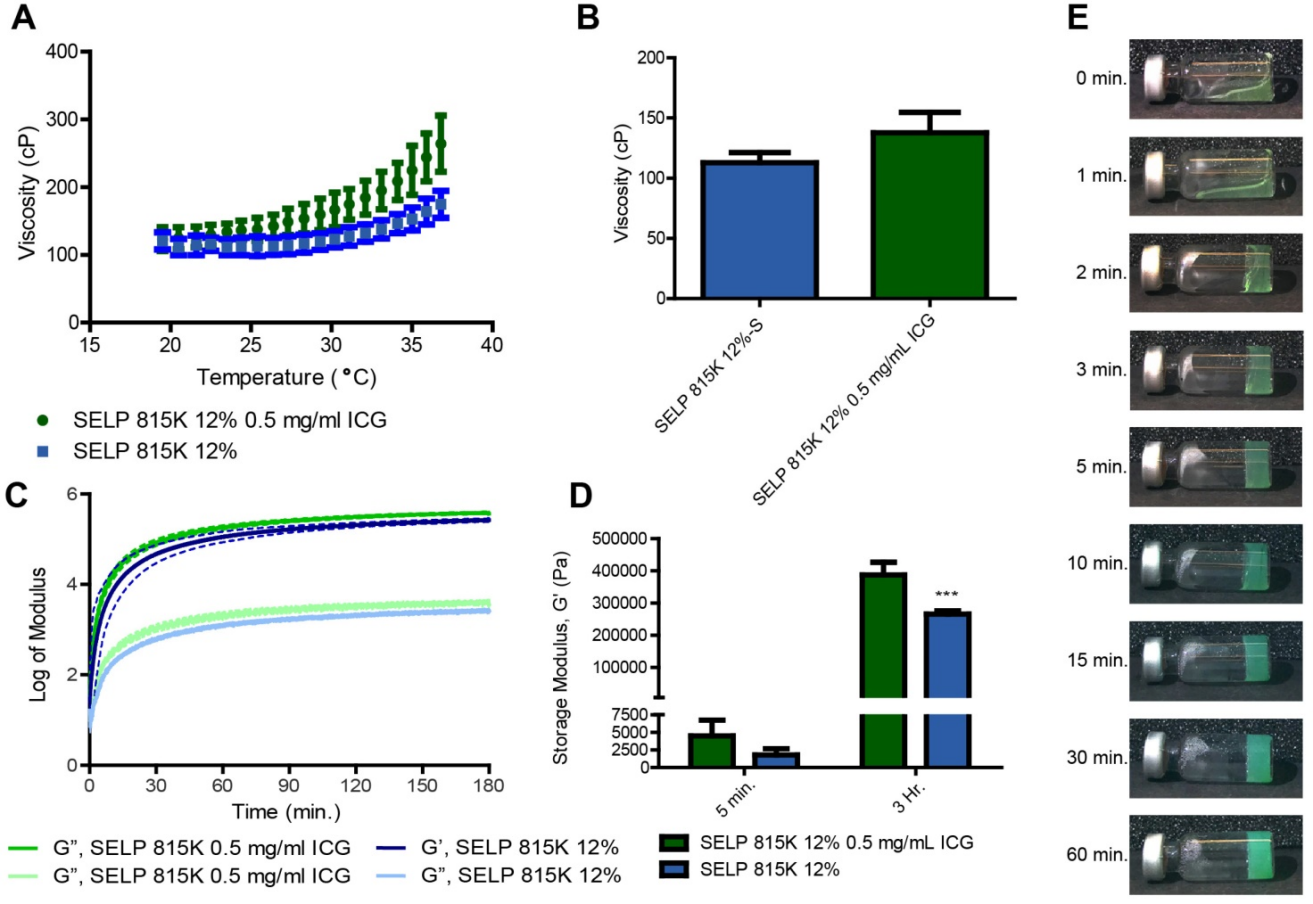
** SELP-ICG viscoelastic properties. A)** Viscosity traces of two embolic formulations from 18-37°C, illustrating that temperature increases SELP viscosity and the addition of ICG enhances this effect. **B)** SELP and SELP-ICG viscosity at 25 °C. **C)** The storage (G') and loss (G”) moduli of SELP and SELP-ICG over a 3-hr period demonstrate rapid gelation kinetics and the formation of a robust gel. The dashed lines indicate the 95% confidence interval. **D)** Storage moduli at 5 min. and 3 hr. show that ICG incorporation increased the strength of the gel. **E)** Tilt test of SELP 815K 12% (wt/wt) with 0.5 mg/mL of ICG at various times at 37 °C. ***p<0.001. The data represent the mean ± st. dev. (n=3).

The incorporation of ICG increased the degree of thermal viscoelastic response of SELP embolic solutions. Initially, the difference between SELP and SELP-ICG was negligible at 18°C (120 ± 13 cP and 123 ± 17 cP, respectively). However, as the samples warmed, ICG incorporation accelerated the temperature-induced increase in SELP viscosity. At 37°C, the viscosity of SELP and SELP-ICG increased to 175 ± 19 cP and 264 ± 42 cP, respectively (Figure 4A). This behavior indicates a 261% increase in the magnitude of the temperature-induced viscosity enhancement from 18 to 37°C for SELP, due to ICG incorporation. However, below room temperature, the viscosities remain low enough for easy injection (Figure 4B). ICG incorporation additionally increased the gelation kinetics and peak strength of the SELP embolic. The slope elevation of the storage modulus during an oscillatory time sweep at 37°C indicates faster gelation (Figure 4C). The 45.9% increase in SELP modulus due to ICG incorporation was highly significant (p<0.001, Figure 4D). Within 2 min at 37°C, SELP-ICG formed a network capable of resisting gravitationally-induced flow. A gradual increase in opacity with time indicates the continuing formation of microdomains within SELP structure that are scattering light and increasing the modulus of the material (Figure 4E). No macroscopic phase was observed, indicating that the transition from an injectable liquid to an occlusive solid was isovolumetric, which is optimal for embolization.

### Release and distribution of ICG in a tissue phantom

**Figure 5:**
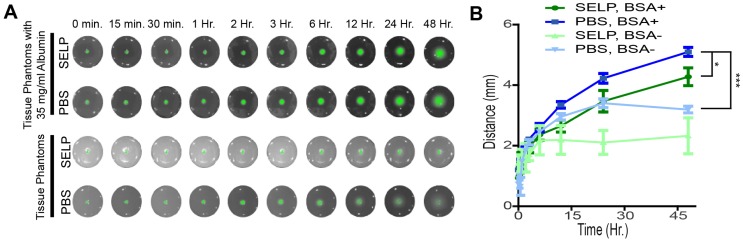
** ICG release and diffusion in agar phantom tissues. A)** ICG fluorescence in tissue phantoms shows release and diffusion after simulated embolization. **B)** BSA enhanced the release of ICG and facilitated diffusion within the tissue phantom and improved fluorescent signal, partitioning from SELP into the phantom. The data points represent the mean ± st. dev. of 6 samples. Comparisons were made between two groups using 2-tailed students T-test of the points at 48 hours. * p<0.05 and *** p<0.001 between the indicated groups.

To fluorescently demarcate tumor margins, ICG must be released from SELP and diffuse into the tissues following embolization of the target vasculature. SELP-ICG was easily injected into tissue phantoms that simulated endovascular embolic delivery and subsequent release of ICG into the surrounding tissue. PBS was held within the channel by sealing the end prior to administration, which would correspond to embolization with a particle-based system immediately following ICG injection via a microcatheter. The incorporation of BSA into the phantom significantly increased both the relative intensity of the ICG and the distance by which ICG diffused within the phantom (Figure 5A). Within 24 hrs after SELP delivery, ICG had diffused to a depth of 3.5 ± 0.7 mm and 4.2 ± 0.4 mm, respectively. In the phantoms without BSA, the ICG had only diffused to a depth of 2.1 ± 0.6 mm, whereas PBS was measured at 3.4 ± 0.4 mm. The difference between SELP with and without BSA is greater than that observed with PBS. Albumin enhanced diffusion and release of ICG from SELP within tissue phantoms. SELP reduced the distance ICG diffused likely by restricting the rate at which the dye was able to partition into the phantoms.

### Imaging of ICG with preclinical tools correlates with clinically available endoscopes

**Figure 6:**
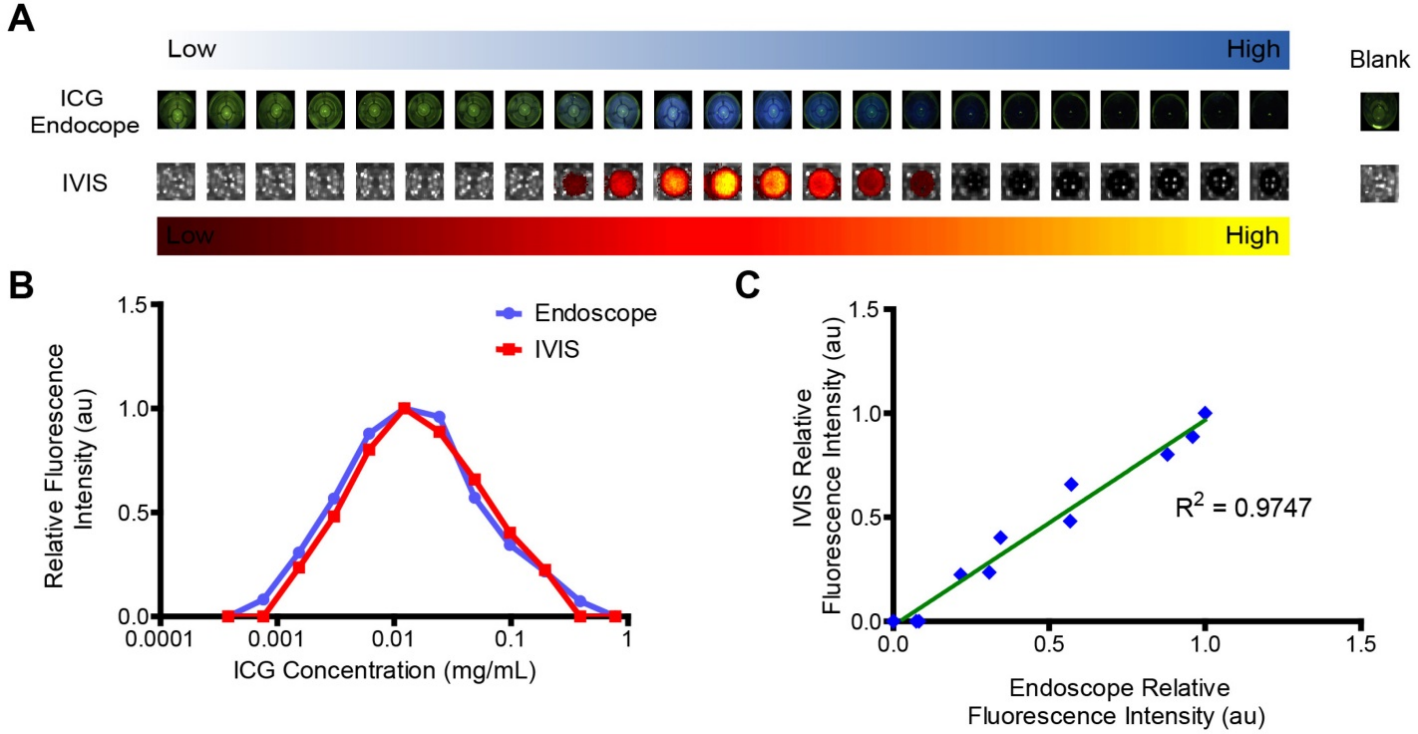
** Visualization of ICG fluorescence. A)** ICG is readily visualized with a commercially available endoscope, shown as blue overlay, and using IVIS preclinical imaging system, shown in a yellow-hot overlay. However, ICG fluorescence dose not directly correlate with concentration. Each well represents a 2x increase in concentration from left to right. **B)** Image analysis demonstrates that there is visually apparent self-quenching that occurs at concentrations higher than 0.012 mg/mL ICG. C) Signal from IVIS and the endoscope are directly proportional.

Imaging on IVIS correlates with imaging findings with a clinical endoscope system. The quenching effects of the ICG can clearly be seen in the images from both IVIS and the Karl Storz ICG endoscope (Figure 6A). The optimal concentration of ICG was 0.012 mg/mL for both systems (Figure 6B). Fluorescence intensity between the two systems was linearly correlated (Figure 6C). The excitation light used by the endoscope gave the wells a green cast. At high concentrations of ICG, this light was absorbed by the ICG, resulting in darker wells. This confirms that IVIS is a capable tool for being able to assess the potential utility of ICG delivery systems for use with the Karl Storz ICG endoscope.

### Embolization in a microfluidic model of tumor vasculature

**Figure 7:**
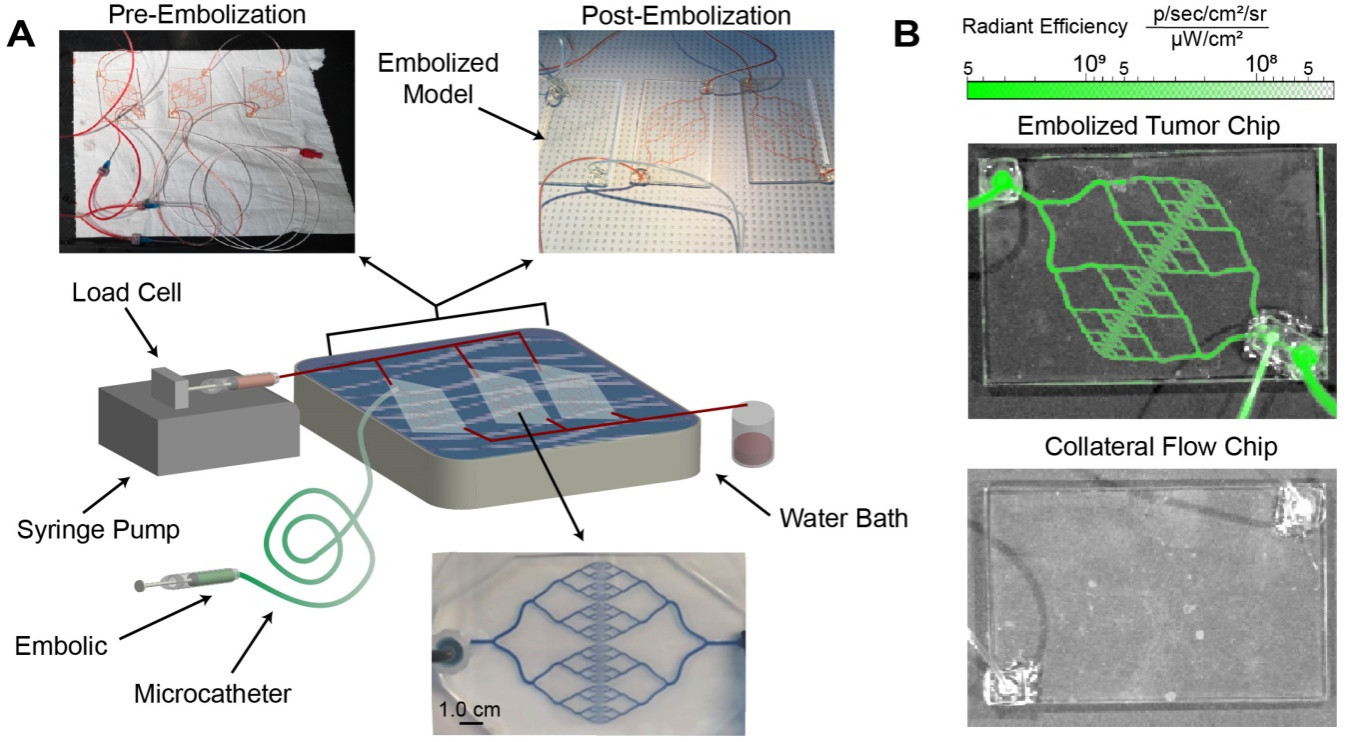
** SELP-ICG embolization and visualization in a microfluidic model tumor. A)** Graphical illustration of embolization test setup with images of microfluidic models before and after embolization. After embolization, there was no perfusion to the embolized chip. Below the illustration is a magnified version of one of the collateral flow chips filled with methylene blue to show channel structure. **B)** IVIS images of the tumor microfluidic chip (top) and a collateral flow chip (bottom) show fluoresce within the SELP-ICG embolized tumor chip but not in the collateral chips.

Local delivery via microcatheter and selective vasculature occlusion are essential features of embolic devices. The embolic capability of SELP-ICG was tested using custom-made, microfluidic tumor vasculature models with clinical microcatheters for simulating the anticipated implementation of the device (see Figure 7A). The microfluidic models went through several phases of design to minimize potential dead space (see Figures S2-S3). The flow through resistance was 0.052 mmHg*min/L for 3 devices in parallel compared to 0.105 mmHg*min/L for a single vascular model. These resistances are not typical in human vascular beds, suggesting that the models herein are at least as challenging if not more so than *in vivo* vasculature of equivalent size. SELP-ICG was injected through a 2.3-Fr, 110-cm catheter submerged in a 37°C water bath. SELP-ICG immediately occluded the vasculature upon reaching the device and redirected flow to the two collateral devices. Fluorescent imaging of the embolized device showed deep penetration and thorough occlusion of the entire device with no evidence of blockage or fluorescence in non-target vascular models (Figure 7B). These results were reproduced in three independent replications for the test embolization. SELP-ICG was able to effectively deliver ICG deep into the vasculature of a microfluidic model tumor, after successfully occluding flow.

### Cytotoxicity of SELP ICG

**Figure 8:**
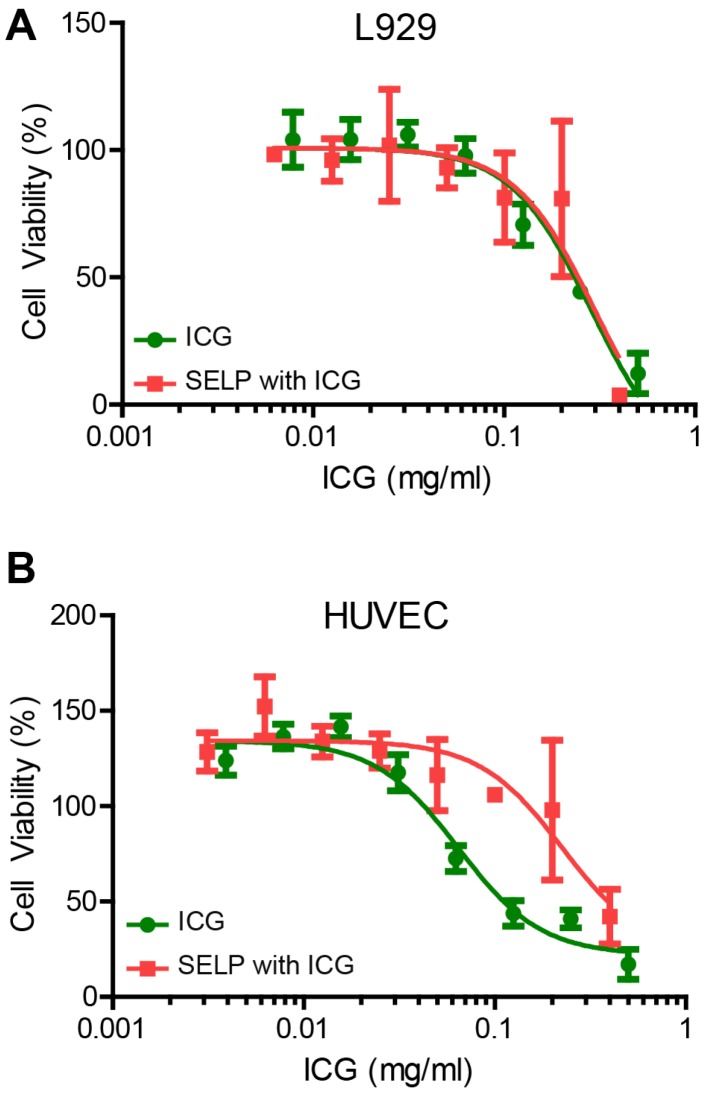
** Cytotoxicity of ICG and SELP-ICG. A)** L929 fibroblast and **B)** HUVEC viability curves in response to increasing ICG concentration of ICG alone or SELP-ICG. The data represent the mean ± st. dev. of 6 samples. The solid lines represent the curve derived from fitting the data to a variable slope Hill equation.

ICG cytotoxicity is ameliorated by SELP for HUVECs but not L929 fibroblasts. ICG is clinically used, but delivering a relatively high concentration locally can negatively impact cells. CCK-8 assay was used to assess the relative viability and health of the cells based on the reduction of a tetrazolium salt by dehydrogenase enzymes via electron mediators, such as nicotinamide adenine dinucleotide (NAD). The LD_50_ for L929 cells was not significantly different for ICG or ICG in SELP, 0.28 ± 0.14 mg/mL and 0.30 ± 0.11 mg/mL, respectively (Figure 8A). However, the LD_50_ for ICG and ICG in SELP in HUVEC was very highly significant with respective values of 0.068 ± 0.009 mg/mL and 0.23 ± 0.03 mg/mL (Figure 8B). In both cases, the addition of SELP either made no difference or ameliorated the toxic effects of ICG. This indicates that ICG incorporation into a hydrogel embolic is potentially feasible from a biocompatibility perspective.

## Discussion

Achieving effective and efficient surgical resection of hypervascular tumors in the head and neck, such as JNAs, is extremely challenging due to the loss of haptic feedback. Bleeding rapidly obscures the endoscopic visual field, which increases the risk of surgical complications while reducing optimal surgical outcomes. The delineation of the boundary between tumor and healthy tissues can also be challenging, especially in the sinonasal cavity where margins are extremely difficult to obtain due to the proximity of critical anatomy within millimeters of the tumor. We hypothesized that the development of an embolic system that delivers a tumor-selective dye can aid surgeons by overcoming the loss of haptic feedback by reducing intraoperative bleeding while visually demarcating tumor boundaries. While numerous embolic systems and strategies have been explored, to our knowledge, no methods have reported the potential synergy between neoadjuvant tumor hemostasis from embolization and fluorescence-based image-guided surgery.

Effective drug delivery requires the precise delivery of the therapeutic agent with respect to location and time. In the context of fluorescence-based image-guided surgery, this means achieving high visual contrast by a localized dye within the tumor and minimizing dye diffusion to the surrounding healthy tissues. Due to its rapid clearance from the bloodstream (3-5 min half-life), free ICG has a limited opportunity to accumulate in the tumor vasculature 14,27. Incorporation of ICG into nanoparticle formulations extends circulation time and can increase accumulation 27-30. However, bypassing the circulation phase of ICG accumulation entirely can elevate the local concentration of ICG beyond that which is achievable with freely circulating molecules.

The tunable biophysical properties of SELP embolics demonstrate promise as delivery vehicles for ICG to tumors. Release over an 18-24 hr. period is desirable for current neoadjuvant embolization practices for JNAs, which are typically embolized a day prior to surgery. Highly vascularized tumors have a reported average distance of 300-350 μm between blood vessels 31. We have shown that the 24 hr. diffusional distance of ICG released from SELP (Figure 5) exceeds the inter-capillary distances within tumors (300-350 μm) allowing complete saturation of the tumor volume. The delivery of 0.5 mg/mL of ICG also means that after 24 hr. of release, the concentration of dye within the tumor will achieve the near optimal concentration for maximizing fluorescent signal and avoid self-quenching effects (Figure 6). This ability to concentrate at 24 hr. is clinically relevant as JNA tumors are typically embolized 24 hours prior to surgical resection. However, further work to characterize diffusion in tissues is needed to verify adequate distribution of ICG within both tumor and healthy tissues.

The SELP-ICG embolic formulation has the potential to also achieve distinct, fluorescently defined tumor margins that can be identified during fluorescence-based image-guided surgery. ICG has been shown in numerous human trials to preferentially accumulate within various types of solid tumors 28,32,33. These phenomena may be attributed to compromised lymphatic drainage in the malignant tissue, which in turn slows ICG clearance when compared to normal tissues 32,33. Delivering higher concentrations of ICG intratumorally via endovascular embolization can potentially further enhance accumulation, as the SELP embolic occludes tumor vessels and prevents ICG clearance by re-entry into tumor vessels. Clearance from the surrounding healthy tissue is unaffected and thus continues to produce a gradient of ICG at the tumor margin. This hypothesis will need to be tested using endovascular administration of ICG in combination with embolization in an *in vivo* model system.

The incorporation of ICG into SELP increased gelation kinetics and stiffness of SELP embolics. Polymer-polymer, polymer-solution, and polymer-solute interactions play a role in this behavior. SELP naturally gels as the silklike-blocks within the back bone form crystalline beat sheet structures that cross link the polymer 34-36. The efficiency of silk-silk interactions is mediated by the relative solubility of the elastinlike blocks within the polymer backbone. As the motion and favorability of the elastinlike blocks is decreases the rate at which silk-like crosslinks form is dramatically enhanced. The addition of ICG increased the osmolarity of the solutions, which has previously been shown to accelerate network formation by increasing the relative favorability of polymer-polymer interactions 20,23. However, these observations alone do not explain the degree of enhancement seen in the viscosity profile. The divalent anionic character of ICG likely created additional bridging interactions between the positively charged lysine residues found within the elastinlike blocks of the SELP polymer backbone. At low temperatures, the polymers were sufficiently soluble such that their Brownian motion rendered SELP-ICG and silk-silk interactions transient. As temperature rose and the relative solubility of the elastinlike-portions of the polymers decreased, the relative strength of the bridging-interactions increased, which led to the observed increase in viscosity and acceleration of gelation.

Concentration, processing, local environment, and structure are the key features that contribute to the gelation and nano- and microscale formation behaviors of SELP 17,22,37-39. SELP penetrated into the venous outflow of the model while being rapidly and substantially diluted. The dilution prevented SELP from forming a cohesive network and the resulting soluble polymers were non-occlusive 17,18,38. SELP 815K, as was used in this study, does not gel below 2% (wt/wt) even after 24 hours at 37°C 38. SELPs at concentrations below 2% form non-occlusive nanostructures ranging from fibers, and spherical nanogels to globular single strand proteins, depending upon environmental conditions 17,40. SELP injection at 0.1 mL/min into our simulated vascular beds, which were perfused at 38 mL/min, represents a 1/380 volumetric dilution. Therefore, the injection under the test conditions represents over a 60× safety margin. This property is similar to that of other clinically used embolic systems, such as LeGoo®, a poly(ethylene glycol)-polyethylene copolymer-based embolic gel, which passes into venous vasculature after producing a transient embolization 7,9. Further testing is needed to establish the safe limits for injection and assess the risk of any downstream embolization events with diluted SELP embolics.

SELP embolic defers from other liquid embolic materials reported in the literature by having a very low initial viscosity (similar to that of contrast agents), ability to irreversibly form a durable crosslinked structure without undergoing any chemical reactions, non-adhesive to clinical catheters, and is chemically with any clinically used catheters. The viscosity of SELP embolic is only 120 ± 13 cP compared to shear thinking biomaterial 4513000 ± 1062000 cP embolic system 41, Onyx 500 is ≈2250 cP 9, poly(ε‐caprolactone)- poly(ethylene glycol)-sulfamethazine under basic conditions is reported as <1000-2500 cP (≈3,900,000 cP under physiological conditions) 9,42, and ≈397,000 cp for coacervation based materials like Sal-IP_6_ 43. High viscosity doesn't preclude these materials from embolic usage but restricts their usage to larger catheters and in peripheral applications usage where arteries are easier to access less selective embolization is acceptable. High viscosity also limits their ability to penetrate deeply into the vascular nidus of hypervascular tumors. Other liquid embolic materials, with low initial viscosity, undergo chemical reactions that can interfere with chemical payloads such as those that use cyanoacrylate chemistry or Schiff base reactions 7,9,44. Particle based embolics accumulate in the peritumoral vasculature and can accumulate at distances greater than a centimeter form the tumor boundary 45. For these reasons a liquid based embolic with a low injection viscosity is an optimal choice for delivering visualization agents deep into tumor vasculature. After penetration of the vascular nidus an ideal embolic must be retained within the tumor to locally deliver its payload. SELP does this by transitioning from a liquid solution to a robust gel that is retained within vasculature capable of resisting intravesicular forces.

The deliverability of SELP embolic was not compromised by the addition of ICG. The difference in viscosity was not significant between SELP and SELP- ICG at temperatures < 25°C. Increased viscosity at 37°C did not interfere with the ability of the SELP to perfuse into a clinically-relevant microfluidic model of tumor vasculature during simulated embolization (Figures 4 & 6). The SELP-ICG embolic was still also able to produce a thorough occlusion. Notably, the flow resistances in the microfluidic models were lower than that measured in tumor tissues, rendering the devices more difficult to embolize (Figure S2) 46. There are several limitations in using these *in vitro* models. These models cannot fully recapitulate the elasticity of vessels, hydrostatic pressure, tortuous three-dimensional geometries, or vascular surface features. Future testing *in vivo* will be essential for accurately assessing the embolic capability and depth of penetration of SELP-ICG into the microvasculature.

Fluorescence visualized in tissue phantoms does not necessarily mirror what will be seen in a clinical setting (Figure 5). Tumor tissues could require higher, or even lower, concentrations of dye to achieve an optimal fluorescent signal. It was also observed that albumin impacted both the release of ICG and the observed intensity of fluorescence (Figure 5). ICG is known to interact with albumin and other globulins 11,12,27,47. The addition of albumin thus likely helps shield the negatively charged ICG from interacting with the positively charged lysines in the SELP backbone. Association with albumin also likely helps disperse ICG within the solution which helps reduce ICG's self-quenching effects (Figures 5 & 6). The auto-quenching effect of ICG can likewise complicate imaging as the intensity decreases, rather than increases, if the concentration of ICG is too high. This threshold appeared to be dependent upon factors, such as volume and geometry, which are difficult to control in a biological environment. As such, the optimal concentration of dye within the embolic cannot be determined without further *in vivo* testing.

A small pilot trial done by Simone and colleagues demonstrated that selective endovascular administration of ICG was able to improve both tumor identification and tumor margins 48. ICG combined with ethiodized oil improved dye retention in target vasculature in endophytic renal masses, allowing surgeons to preserve healthy parenchyma and reduced the risk of complication. Although, the findings were not statistically significant due to the limited size of the cohort, the authors did demonstrate potential for using endovascular techniques to fluorescently demarcate tumors to improve surgical resection. The tool developed in this manuscript seeks to carry the concept of endovascular tumor demarcation even further, by also leveraging embolization to reduce intraoperative bleeding which can additionally improve visualization both under fluorescent and standard lighting conditions.

The dual-functional embolization-visualization system, based upon SELP embolics and near IR dye ICG, was developed and characterized for future clinical application in combining embolotherapy with fluorescence-based image-guided surgery. Many of the strategies developed as part of this work could be additionally explored with clinically used materials. Similar to conventional transarterial chemoembolization procedures, ICG could be deployed intravascularly and immediately followed by embolization with a particle based-embolic 7,20,49. While this technique would allow for the assessment of ICG with embolotherapy using current clinical materials, the system developed herein offers potential clinical advantages over other materials. Controlled localized release of the fluorescent marker directly from the SELP embolic might enhance tumor demarcation contrast by increasing the intratumoral load, reducing intratumoral clearance, and reducing the amount of dye that enters healthy tissues.

## Conclusions

We illustrated that ICG incorporation into and release from SELP embolic materials is feasible. ICG-polymer interactions increase the viscosity, accelerate gelation, and increase the stiffness of the SELP embolics. ICG is deliverable from SELP over a clinically pertinent time frame. Combining embolization with delivery of a fluorescent dye to hypervascular tumors offers an opportunity to improve surgical visualization by reducing intraoperative bleeding, while simultaneously demarcating tumor margins.

## Supplementary Material

Supplementary figures.

## Abbreviations

ANOVA: analysis of variance
ATCC: American type culture collection
cm: centimeter
BSA: bovine serum albumin
DMEM:F12: Dulbecco's modified eagle medium/nutrient mixture f-12
ECGM: endothelial cell growth medium
FBS: fetal bovine serum
Fr: French gauge system
g: gram
G': storage modulus
G”: loss modulus
hr: hour
HUVEC: human umbilical vein endothelial cells
ICG: indocyanine green
IVIS: in vivo imaging system
JNA: juvenile nasopharyngeal angiofibroma
LD50: lethal dose, 50%
mg: milligram
min: minute
mL: milliliter
mm: millimeter
NIR: near infrared
Pa: pascal
cP: centipoise
PBS: Phosphate Buffered Saline
SELP: silk-elastinlike protein
SEM: scanning electron microscopy
st. dev.: standard deviation
µL: microliter
wt: weight
