# Supplementary material for: A dual-functional Embolization-Visualization System for Fluorescence image-guided Tumor Resection
Source: Theranostics. 2020 Mar 15;10(10):4530–43. doi: 10.7150/thno.39700 (PMC7150499; doi:10.7150/thno.39700)
Supplement: Supplementary file 1 — Supplementary figures. [file thnov10p4530s1.pdf]

A Dual-Functional Embolization-Visualization System for Fluorescence Image-Guided Tumor Resection

M. Martin Jensen[a,b] Zachary B. Barber[a,b], Nitish Khurana[b,c], Kyle J. Isaacson[a,b], Douglas Steinhauff[a,b], Bryant Green[a,b], Joseph Cappello[c], Abigail Pulsipher[b,d], Hamidreza Ghandehari[a,b,c,d†], Jeremiah A. Alt[a,b,c,d†]

[a]Department of Bioengineering, University of Utah, Salt Lake City, UT, 84112 USA
[b]Utah Center for Nanomedicine, Nano Institute of Utah, University of Utah, Salt Lake City, UT, 84112 USA
[c]Department of Pharmaceutics and Pharmaceutical Chemistry, University of Utah, Salt Lake City, UT, 84112 USA
[d]Department of Surgery, Division of Otolaryngology–Head and Neck Surgery, University of Utah School of Medicine, Salt Lake City, UT 84113

†Co-corresponding Authors:

Hamidreza Ghandehari, PhD
University of Utah
36 S. Wasatch Dr.
Sorenson Molecular Biotechnology Building, 5211
Salt Lake City, UT 84112-5001
hamid.ghandehari@utah.edu

Jeremiah Alt, MD, PhD
University of Utah
50 North Medical Drive
School of Medicine, 3C120
Salt Lake City, UT 84132
jeremiah.alt@hsc.utah.edu

Supplemental Information

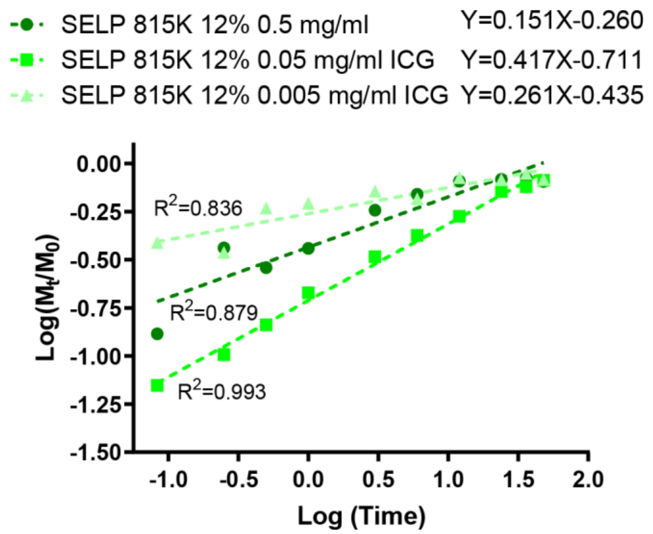

Figure S1: Modeling of ICG release from SELP matrices using the Korsmeyer-Peppas model. Points represent the average of 6 experimental replicates. The doted lines represent the regression line for each data set.

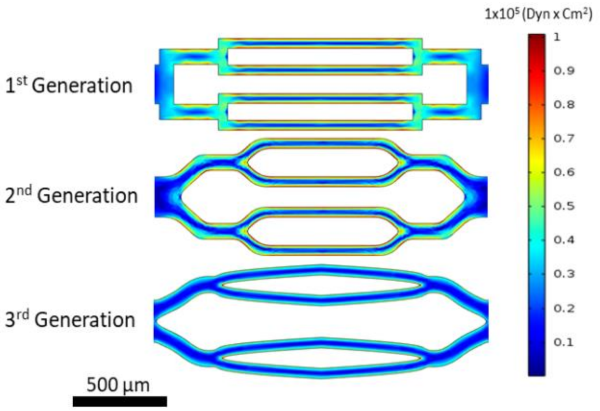

Figure S2: Computational modeling of shear force of simulated blood flowing through microfluidic tumor models. Color gradient represents the shear force experienced by the fluid for **A)** 1st Generation, **D)** 2nd Generation, and **C)** 3rd Generation designs. Images and models were generated using Comsol Multiphysics 5.4. The designs were developed to reduce turbulent flow and reduce dead space within the structures.

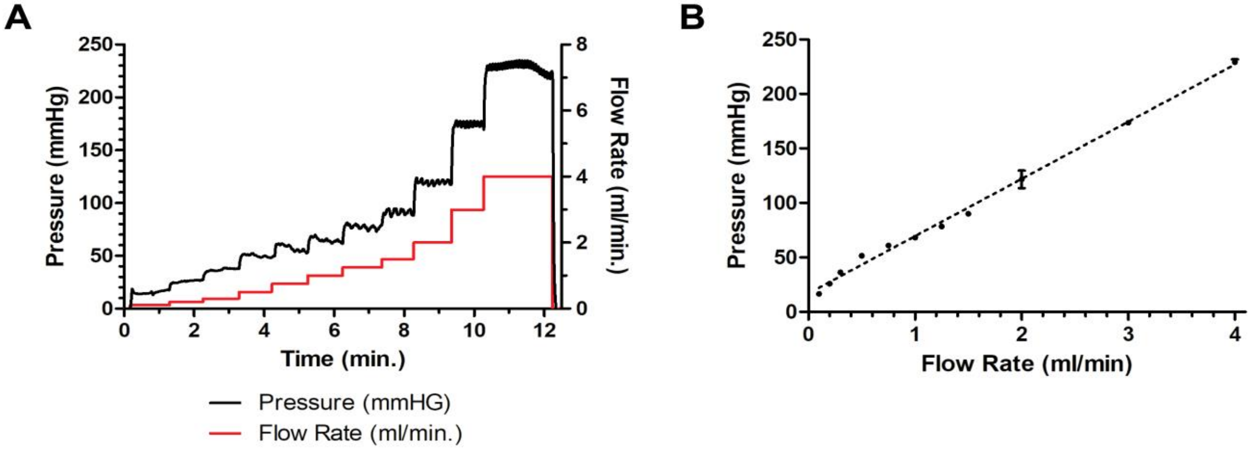

Figure S3: Pressure vs. flow rate through 3 microfluidic tumor models plumbed in parallel. A) Pressure profiles with a flow rate ramp using PBS for the 3rd generation design of microfluidic tumor model. **B)** Flow rate vs. pressure showed the anticipated linear relationship. The dashed line indicates the regression line of the flow profile. Each point represents the average of 10 seconds of data taken form the equilibrium pressure of the system at each flow rate.
